# The efficacy and safety of ALK inhibitors in the treatment of ALK‐positive non‐small cell lung cancer: A network meta‐analysis

**DOI:** 10.1002/cam4.1768

**Published:** 2018-09-19

**Authors:** Junsheng Fan, Tszhei Fong, Zengfei Xia, Jian Zhang, Peng Luo

**Affiliations:** ^1^ Department of Oncology Zhujiang Hospital of Southern Medical University Guangzhou China; ^2^ Department of Respiratory Medicine Shanghai Tenth People's Hospital Tongji University Shanghai China

**Keywords:** anaplastic lymphoma kinase inhibitor (ALKi), brain metastasis, network meta‐analysis, non‐small cell lung cancer (NSCLC)

## Abstract

**Purpose:**

The current study was carried out to compare the effectiveness and safety of different ALK inhibitors in treating ALK+ NSCLC.

**Methods:**

Progression‐free survival (PFS), disease control rate (DCR), overall response rate (ORR), and intracranial ORR and DCR have been aggregated to appraise the effectiveness of each ALKi. The discontinuation rate due to adverse events (AEs) was pooled to evaluate their safety. Bayesian network meta‐analyses were used to compare the ORR, DCR, PFS, and discontinuation rate of patients treated with alectinib, ceritinib, crizotinib, and chemotherapy.

**Results:**

Compared with chemotherapy, ALK inhibitors significantly prolonged PFS [hazard ratio (HR) and 95% confidence interval (CI): alectinib, 0.50 (0.43‐0.58); ceritinib, 0.75 (0.69‐0.83); crizotinib, 0.71 (0.66‐0.76)]. The ORRs were significantly higher for ALK inhibitors than for chemotherapy [odds ratio (OR) and corresponding 95% CI: alectinib, 11.69 (4.29‐36.56); ceritinib, 7.85 (3.44‐19.27); crizotinib, 6.04 (3.33‐11.71)]. The discontinuation rates were lower for ALK inhibitors than for chemotherapy [OR and corresponding 95% CI: alectinib, 0.42 (0.12‐1.36); ceritinib, 0.52 (0.20‐1.35); crizotinib, 0.70 (0.30‐1.62)].

**Conclusions:**

ALK+ NSCLC patients treated with ALKi tend to have longer PFS than those treated with chemotherapy. ALKi‐naïve patients tended to response better than their ALKi‐pretreated counterparts. Alectinib appeared to be preferable for treating brain metastases due to its high intracranial efficacy. Patients treated with alectinib or ceritinib tended to have higher ORR and DCR than patients with similar baselines treated with crizotinib or chemotherapy. No significant differences in discontinuation rate were found for alectinib, ceritinib, crizotinib, and chemotherapy.

## 1 INTRODUCTION

The current incidence of lung cancer continues to increase due to widespread risk factors, such as cigarette smoking and air pollution.^1^ Consequently, lung cancer has become the leading cause of cancer‐related mortality worldwide.^1^ Non‐small cell lung cancer (NSCLC) and small cell lung cancer are two classifications of lung cancer. Importantly, according to its histopathological features, NSCLC accounts for nearly 85% of all cases of lung cancer.^1^ Chemotherapy is a common method for treating NSCLC, but it has limited survival benefits and considerable adverse effects, including alopecia, dyspnea, and neutropenia. Thus, molecular targeted therapies with high specificity are urgently needed.^2^, ^3^, ^4^, ^5^

Anaplastic lymphoma kinase (ALK), which is expressed highly in the mammalian nervous system, is a therapeutic target for the treatment of NSCLC.^6^ Moreover, the echinoderm microtubule‐associated protein‐like 4 (EML4)‐ALK fusion protein is a type of ALK fusion protein that plays an important role in tumorigenesis in approximately 5% of NSCLC cases.^7^ The first FDA‐approved ALK inhibitor (ALKi) for the treatment of NSCLC with ALK fusion proteins (ALK‐positive, ALK+) is an aminopyridine compound, crizotinib. Crizotinib can inhibit ALK, hepatocyte growth factor receptor protein‐tyrosine kinase, and ROS proto‐oncogene 1 (ROS1) receptor tyrosine kinase.^7^ However, crizotinib resistance develops in approximately 60% of patients after 10.5 months of treatment. Therefore, several second‐generation ALK inhibitors, including ceritinib and alectinib, have been developed to avoid crizotinib resistance.^7^, ^8^

However, direct head‐to‐head clinical trials of the efficacy and safety of different ALK inhibitors are limited.^9^, ^10^ Only four meta‐analysis studies have analyzed the efficacy and safety of ALKi (two studies on crizotinib, one on alectinib and one on overall ALKi) for treating ALK‐positive NSCLC.^11^, ^12^, ^13^, ^14^ Hu et al and Qian et al pooled the progression‐free survival (PFS) and overall response rate (ORR) from 13 clinical trials of NSCLC patients undergoing different lines of treatments with crizotinib. These authors also included ORR, PFS, 1‐year overall survival (OS), complete response, partial response, stable disease, and dose reduction in crizotinib‐treated NSCLC patients from six clinical trials in the meta‐analyses to evaluate the efficacy and safety of crizotinib.^11^, ^12^ Fan et al^13^ not only pooled the efficacy and safety parameters to evaluate alectinib but also included the intracranial ORR. Li et al^14^ reported better outcomes regarding OS, PFS, and ORR in a meta‐analysis of the overall therapeutic outcomes of ALKi. Nevertheless, these previous meta‐analyses do not include the present clinical trials, which have recently been published; thus, updates are necessary. In our study, we included 33 clinical trials, including eight randomized controlled trials (RCTs), in a Bayesian network meta‐analysis. We also included all ALKi arms of RCTs and 25 single‐arm trials in single‐arm meta‐analyses. For these single‐arm studies, the ORR and PFS serve as the summary measures. In addition, alectinib, ceritinib, and crizotinib were compared with chemotherapy in a network meta‐analysis, and the hazard ratios (HRs) were pooled.

## 2 MATERIALS AND METHODS

### 2.1 Search strategy

The current study was carried out in accordance with the PRISMA statement (preferred reporting items for systematic reviews and meta‐analysis) as well as the PRISMA extension statement for network meta‐analyses to obtain the least biased evidence for clinical practice.^15^, ^16^

We searched three databases (PubMed, the Cochrane Library and Web of Science) on 1 March 2018. All the keywords (NSCLC, ALK inhibitors) as well as their MeSH terms and entry terms have been used to build our search strategies.

For example, the following search strategy was used for the Cochrane Library: (“Non‐Small Cell Lung Cancer” OR “Non‐Small Cell Lung Carcinoma” OR “Non Small Cell Lung Carcinoma” OR “Non‐Small‐Cell Lung Carcinoma” OR “Nonsmall Cell Lung Cancer” OR “Non‐Small‐Cell Lung Carcinomas” OR “Non‐Small‐Cell Lung Carcinoma” OR “NSCLC”) AND (“Crizotinib” OR “PF‐02341066” OR “Xalkori” OR “alectinib” OR “Alecensa” OR “RO5424802” OR “CH5424802” OR “Ceritinib” OR “LDK378” OR “Zykadia” OR “Brigatinib” OR “AP26873” OR “lorlatinib” OR “PF‐06463922” OR “Entrectinib” OR “RXDX‐101” OR “NMS‐E628” OR “ASP3026” OR “Ensartinib” OR “X‐396” OR “TSR011” OR “CEP‐37440” OR “KRCA‐0080” OR “TAE684” OR “NVP‐TAE684” OR “AP26113” OR “AZD3463” OR “CM‐118”).

We have also inspected the reference list of the retrieved studies in case we would miss relevant studies which met our inclusion criteria. Additionally, we attempted to contact the corresponding authors by email if there was not enough information about a study in the databases. We also screened the abstract books of the World Conference on Lung Cancer (IASLC WCLC), the annual congress of Asian Pacific Society of Respirology (APSR), the European Respiratory Society (ERS) international congress, the American Society of Clinical Oncology (ASCO) annual meeting, and the American Thoracic Society (ATS) international conference in the past 3 years so that we would not miss the latest progress and results of ongoing clinical trials.^17^, ^18^, ^19^, ^20^, ^21^, ^22^, ^23^, ^24^, ^25^, ^26^, ^27^, ^28^, ^29^, ^30^, ^31^

### 2.2 Study selection criteria

Here are the inclusion criteria: (a) types of studies: clinical trials; (b) participants: NSCLC harboring ALK rearrangement; (c) interventions: ALK inhibitors (alectinib, brigatinib, ceritinib, crizotinib, etc.); (d) outcome measures: PFS and/or ORR; and (e) accessible full text.

Studies that met the following criteria were excluded: duplicate publications, literature reviews, systematic reviews, case reports or case series, animal experiments, cell experiments, or unavailable outcome measures.

### 2.3 Quality assessment of included studies

All the included studies were evaluated by two reviewers (Junsheng Fan & Tszhei Fong) independently. The Cochrane collaboration risk of bias (ROB) tool was applied to appraise the methodological quality of the included phase 3 clinical trials.^32^ The overall risk of bias of a study was considered “low” if no less than four items were rated as “low risk.” If two or three items were rated as “low risk”, the overall risk of bias was considered “moderate”. The overall risk of bias was considered to be “high” if less than two items were marked as “low risk” or no less than two items were marked as “high risk.” We used the NOS scale (Newcastle‐Ottawa scale)^33^, ^34^, ^35^ to appraise the methodological quality of non‐RCT.^33^, ^34^, ^35^

### 2.4 Data extraction

Two reviewers (Junsheng Fan & Tszhei Fong) evaluated all of the studies independently according to the inclusion and exclusion criteria. With a data extraction template designed beforehand, two reviewers collected these data, respectively: the first author's name, study design, publication year, sample size, intervention and control methods, median PFS, OS, response rate, HR, and 95% confidence interval (CI), as well as the information required to appraise the quality of each study. Any inconsistency during the courses of study selection, quality assessment, and data collecting was settled by discussing with the third reviewer (Peng Luo).

If the necessary data were not provided in the paper, we attempted to measure the Kaplan‐Meier curve using GetData Graph Digitizer 2.26. We also attempted to calculate the required variables according to the protocol developed by Tierney et al^36^, ^37^ whenever a Kaplan‐Meier curve with enough resolution was available. Otherwise, the corresponding author of the published study was contacted to obtain the data required for the analysis.

### 2.5 Statistical analysis

Single‐arm meta‐analyses were performed using STATA 13.0 software (Stata Corp., College Station, TX) and Review Manager5.3 (Cochrane Collaboration, London, United Kindom). For the single‐arm studies, ORR and PFS served directly as the summary measures. Heterogeneity between the studies was assessed with the Chi‐square test and *I* ^2^ statistic. A *P*‐value of greater than 0.1 and an *I* ^2^ value of less than 50% indicated no statistically significant heterogeneity. When this was the case, a fixed‐effects model was employed for the meta‐analysis. For *P*‐values of less than or equal to 0.1 and *I* ^2^ values of greater than or equal to 50%, the inter‐study heterogeneity was too significant to be overlooked, and the random‐effects model was employed. Funnel plots were used to visualize the publication bias. Begg's test and Egger's test were used to appraise publication bias quantitatively.

STATA 13.0 software (Stata Corp.) was used to draw a network plot depicting the geometry of the network. Each node represents a treatment, and the diameter of the node is proportional to the total treatment sample size. The number of head‐to‐head trials can be visualized by the thickness of the line between two nodes.^38^ We combined the odds ratios (ORs) of the binary outcome measures (ORR, DCR, and discontinuation rate) to compare the efficacy and safety of each treatment. Additionally, we pooled the HRs of PFS to compare the effectiveness of each drug at prolonging PFS.

OpenBUGS (version 3.2.3 rev 1012) software was used to perform Bayesian network meta‐analyses. The random‐effects model employing Markov chain Monte Carlo methods was used.^39^, ^40^ We generated four chains and used 50 000 iterations with 20 000 burn‐ins for each chain. The thinning interval was 10. To estimate which treatment is likely to be the best in terms of efficacy and safety, the treatments were ranked by their probability at each ranking position. The Brooks‐Gelman‐Rubin method was used to examine the convergence of iterations. A potential scale reduction factor (PSRF) closer to one indicates better convergence.^41^ To determine whether inconsistency existed, we compared the pooled results of outcome measures from traditional pairwise meta‐analyses and Bayesian meta‐analyses, as well as the results from consistency and inconsistency models of network meta‐analyses.^42^, ^43^

In addition, we extracted survival data from Kaplan‐Meier curves of PFS using GetData Graph Digitizer 2.26 software (http://getdata-graph-digitizer.com) and calculated the event population and the censored population at each time interval using the formulas provided in the study by Tierney et al.^37^ Subsequently, we attempted to generate pooled Kaplan‐Meier curves for the PFS of patients treated with alectinib, ceritinib, crizotinib, and chemotherapy. Log‐rank tests were used to assess the differences between each treatment, with *P* < 0.05 considered statistically significant. Kaplan‐Meier analyses and log‐rank tests were performed using SPSS 20.0 (IBM, Armonk, NY, USA).

## 3 RESULTS

### 3.1 Characteristics of the included studies

**Figure 1:**
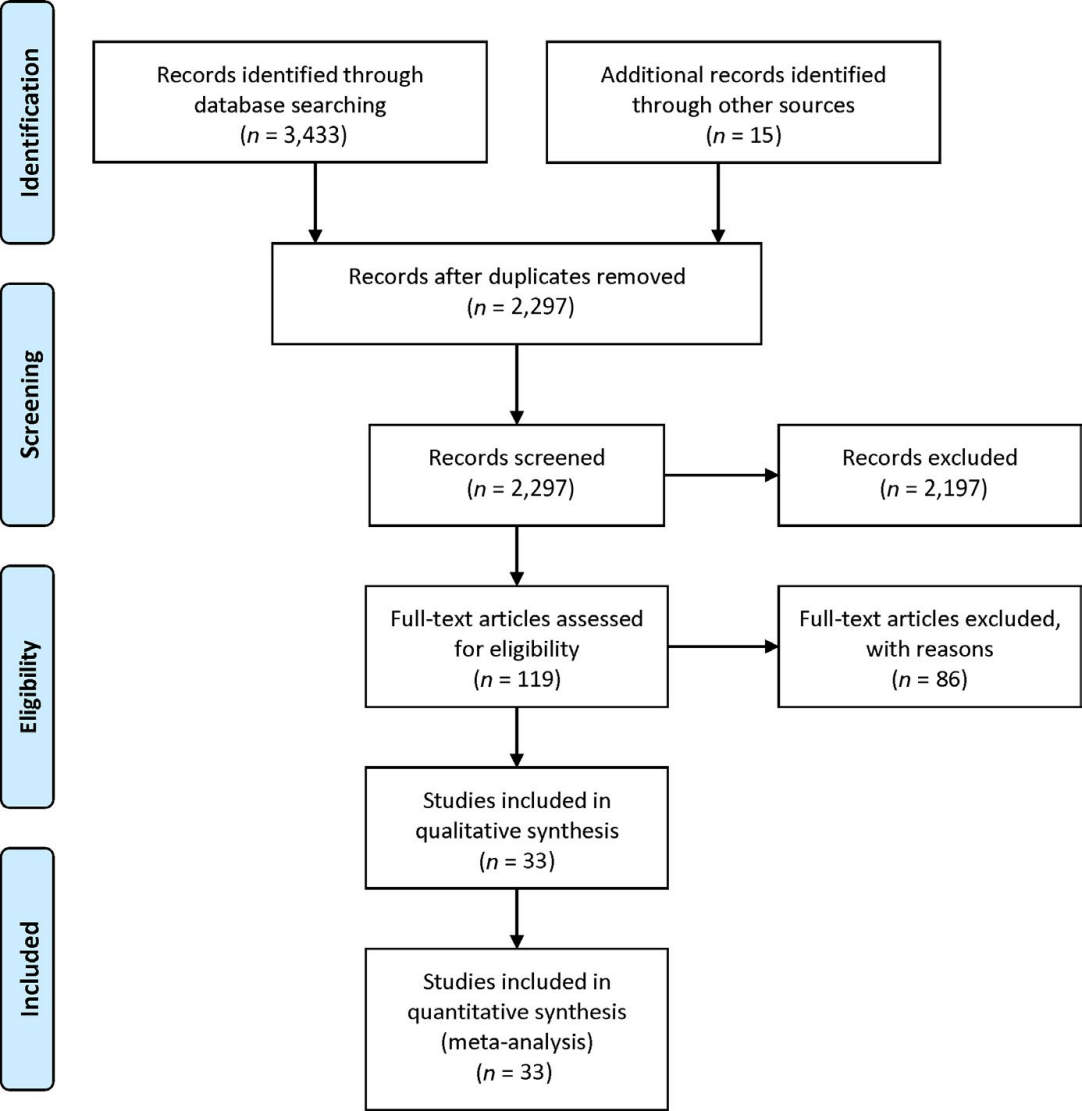
Flow diagram of study selection process

A total of 3433 references have been retrieved from the databases (PubMed: 1171, Cochrane Library: 154, Web of Science: 2108), and 2297 references remained after deduplication. Of these, 2178 references that included preclinical studies, diagnostic trials, case reports or case series, systematic and literature reviews as well as other studies which met the exclusion criteria were excluded. Eventually, 33 clinical studies containing 5507 participants (2042 in the eight RCTs, 3465 in the 25 non‐RCTs) were included (Figure 1). The basic characteristics and quality assessment of the included studies are displayed in Table 1.

**Table 1:** Characteristics of the included studies

Citation	Number of patients	Median age (y)	Median duration of follow‐up (mo)	Treatment	Trial phase	Baseline	Quality assessment (NOS unless Otherwise stated)
Hida 2017 (J‐ALEX)	103	61.0	12.0	ALC 300 mg PO BID	3	ALKi‐naïve	High risk
	104	60.0	12.2	CRZ 250 mg PO BID			
Peters 2017 (ALEX)	152	58.0	18.6	ALC 600 mg PO BID	3	ALKi‐naïve	High risk
	151	54.0	17.6	CRZ 250 mg PO BID			
Soria 2017 (ASCEND‐4)	189	55.0	NA	CER 750 mg PO QD	3	ALKi‐naïve	Low risk
	187	54.0	NA	DDP 75 mg/m^2^ or CBP AUC 5‐6 mg/mL·min + PMX 500 mg/m^2^ IV q3w			
Shaw 2017 (ASCEND‐5)	115	54.0	16.6	CER 750 mg PO QD	3	CRZ‐pretreated	Low risk
	116	54.0	16.4	PMX 500 mg/m^2^ or DTX 75 mg/m^2^ IV q3w			
Shaw 2013 (PROFILE 1007)	173	51.0	12.2	CRZ 250 mg PO BID	3	ALKi‐naïve	Medium risk
	174	49.0	12.1	PMX 500 mg/m^2^ or DTX 75 mg/m^2^ IV q3w			
Solomon 2014 (PROFILE 1014)	172	52.0	10.9	CRZ 250 mg PO BID	3	ALKi‐naïve	Medium risk
	171	54.0	7.0	DDP 75 mg/m^2^ or CBP AUC 5‐6 mg/mL·min + PMX 500 mg/m^2^ IV q3w			
Lu 2016 (NCT01639001)	104	NA	NA	CRZ 250 mg PO BID	3	ALKi‐naïve	Medium risk
	103	NA	NA	DDP 75 mg/m^2^ or CBP AUC 5‐6 mg/mL·min + PMX 500 mg/m^2^ IV q3w			
Zhao 2015	14	55.3	12	CRZ 250 mg PO BID	3	ALKi‐naïve	Medium risk
	14	58.1	12	DTX 75 mg/m^2^ IV q3w			
Seto 2013 (AF‐001JP)	46	48.0	7.6	ALC 300 mg PO BID	1‐2	ALKi‐naïve	6
Gadgeel 2014 (AF‐002JG)	47	56.0	4.2	ALC 300‐900 mg PO BID	1/2	CRZ‐pretreated	3
Hida 2016 (JP28927)	35	45.0	NA	ALC 300 mg PO BID	1/2	Mixed	5
Ou 2016 (NP28673)	138	51.5	7.5	ALC 600 mg PO BID	2	CRZ‐pretreated	6
Shaw 2016 (NP28761)	87	54.0	9.9	ALC 600 mg PO BID	2	CRZ‐pretreated	6
Iwama 2017	18	72.0	9.8	ALC 300 mg PO BID	2	Mixed	3
Kim 2017 (ALTA)	222	54.0	8.0	BRG 90‐180 mg PO QD	2	CRZ‐pretreated	4
Gettinger 2016 (NCT01449461)	79	54.0	17.0	BRG 30‐300 mg PO QD	1/2	Mixed	5
Kim 2016 (ASCEND‐1)	255	53.0	11.1	CER 750 mg PO QD	1	Mixed	6
Crino 2016 (ASCEND‐2)	140	51.0	8.8	CER 750 mg PO QD	2	CRZ‐pretreated	5
Felip 2016 (ASCEND‐3)	124	NA	23.1	CER 750 mg PO QD	2	ALKi‐naïve	3
Zhang 2016 (ASCEND‐6)	103	49.0	8.3	CER 750 mg PO QD	1/2	CRZ‐pretreated	5
Cho 2017 (ASCEND‐8)	137	56.0	4.1	CER 450/600/750 mg PO QD	1	ALKi‐naïve	5
Nishio 2015 (NCT01634763)	20	44.0	NA	CER 300‐750 mg PO QD	1	Mixed	5
Cadranal 2015	155	57.0	NA	CER 750 mg PO QD	1	Mixed	3
Camidge 2012 (NCT00585195)	149	52.0	16.3	CRZ 250 mg PO BID	1	ALKi‐naïve	6
Blackhall 2017 (PROFILE 1005)	1066	52.0	NA	CRZ 250 mg PO BID	2	ALKi‐naïve	6
Cui 2015	72	55.0	NA	CRZ 250 mg PO BID	2	ALKi‐naïve	6
Fujiwara 2016	8	59.0	NA	CRZ 250 mg PO BID	1	ALKi‐naïve	5
Wu 2015	21	51.0	NA	CRZ 250 mg PO BID	2	ALKi‐naïve	3
Camidge 2011	119	51.0	11.0	CRZ 250 mg PO BID	1	ALKi‐naïve	4
Bang 2010	76	NA	NA	CRZ 250 mg PO BID	2	ALKi‐naïve	4
Shaw 2017 (NCT01970865)	41	50.0	17.4	LOR 10‐200 mg PO QD or 35‐100 mg PO BID	1	Mixed	4
Solomon 2017 (NCT01970865)	227	NA	NA	LOR 100 mg PO QD	2	Mixed	3
Horn 2017 (NCT01625234)	80	54.0	NA	ENS 225 mg PO QD	1/2	Mixed	4

ALKi, anaplastic lymphoma kinase inhibitor; ALC, alectinib; AUC, Area under the plasma drug concentration versus time curve; BID, twice a day; BRG, brigatinib; CBP, carboplatin; CER, ceritinib; CRZ, crizotinib; DDP, cisplatin; DTX, docetaxel; ENS, ensartinib; IV, intravenous; LOR, lorlatinib; NA, not available; NOS, Newcastle‐Ottawa Scale; PMX, pemetrexed; PO, take orally; QD, once a day; q3w, every 3 weeks; ROB, risk of bias.

### 3.2 Network meta‐analysis

**Figure 2:**
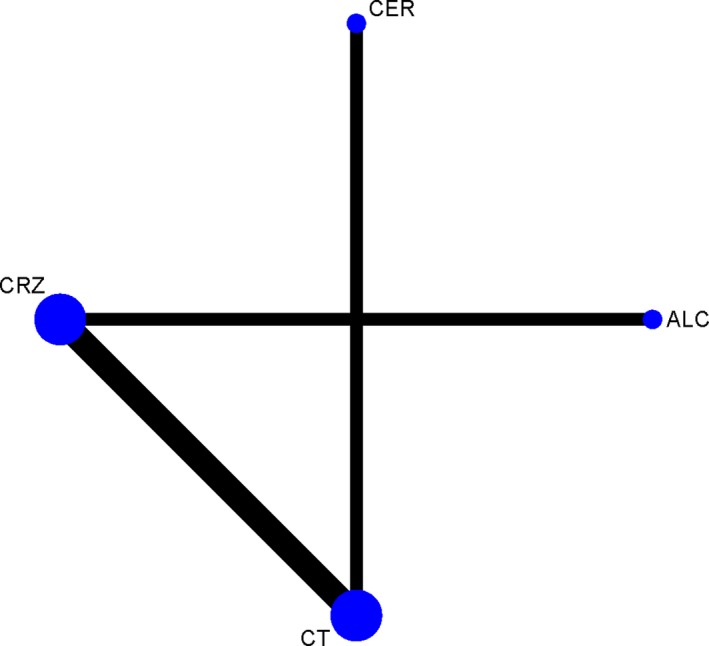
Network of multiple‐treatment comparisons for the Bayesian network meta‐analysis. Each node represented a treatment, the diameter of the node was proportional to the total sample size of a treatment. The number of head‐to‐head trials was visualized by the thickness of the line between two nodes

The structure of the network was displayed in Figure 2. Compared with chemotherapy, ALK inhibitors significantly prolonged PFS [HR and corresponding 95% CI: alectinib, 0.50 (0.43‐0.58); ceritinib, 0.75 (0.69‐0.83); crizotinib, 0.71 (0.66‐0.76)] (Table 2A). Rank probabilities indicated that alectinib is likely to be the best among the four treatments at prolonging PFS. However, crizotinib, rather than ceritinib, may be the second best (Table 3A). Pooled HRs also indicated that the PFS was shorter for ceritinib‐treated patients than for crizotinib‐treated counterparts, although the difference was not statistically significant [HR and corresponding 95% CI: 1.07 (0.95‐1.20)]. Chemotherapy was the worst among them.

**Table 2:** Multiple‐treatment comparisons (MTCs) for efficacy and safety based on network

(A) PFS			
Alectinib			
0.66 (0.56‐0.78)	Ceritinib		
0.70 (0.61‐0.80)	1.07 (0.95‐1.20)	Crizotinib	
0.50 (0.43‐0.58)	0.75 (0.69‐0.83)	0.71 (0.66‐0.76)	Chemotherapy

(B) ORR			
Alectinib			
1.49 (0.39‐5.99)	Ceritinib		
1.94 (0.85‐4.70)	1.31 (0.45‐3.78)	Crizotinib	
11.69 (4.29‐36.56)	7.85 (3.44‐19.27)	6.04 (3.33‐11.71)	Chemotherapy

(C) DCR			
Alectinib			
1.25 (0.05‐53.47)	Ceritinib		
1.87 (0.33‐13.85)	1.48 (0.07‐23.58)	Crizotinib	
7.41 (0.86‐105.72)	5.83 (0.49‐66.28)	3.95 (0.96‐20.61)	Chemotherapy

(D) Discontinuation rate			
Alectinib			
0.81 (0.17‐3.57)	Ceritinib		
0.59 (0.24‐1.39)	0.74 (0.21‐2.68)	Crizotinib	
0.42 (0.12‐1.36)	0.52 (0.20‐1.35)	0.70 (0.30‐1.62)	Chemotherapy

Results in each cell represent the pooled OR/HR and their 95% CI for each outcome measure (the column treatment comparing with the row treatment).

CI, confidence interval; DCR, Disease control rate; HR, Hazard ratio; OR, Odds ratio; ORR, Overall response rate; PFS, progression‐free survival.

**Table 3:** Rank probabilities of each treatment for different outcome measures based on network

Drug	Rank 1	Rank 2	Rank 3	Rank 4
(A) PFS				
Alectinib	1.00	0.00	0.00	0.00
Ceritinib	0.00	0.14	0.86	0.00
Crizotinib	0.00	0.86	0.35	0.00
Chemotherapy	0.00	0.00	0.00	1.00
(B) ORR				
Alectinib	0.76	0.20	0.03	0.00
Ceritinib	0.22	0.52	0.26	0.00
Crizotinib	0.02	0.28	0.70	0.00
Chemotherapy	0.00	0.00	0.00	1.00
(C) DCR				
Alectinib	0.51	0.33	0.13	0.03
Ceritinib	0.41	0.25	0.28	0.06
Crizotinib	0.08	0.41	0.49	0.02
Chemotherapy	0.00	0.01	0.10	0.89
(D) Discontinuation rate				
Alectinib	0.03	0.06	0.30	0.61
Ceritinib	0.06	0.23	0.34	0.36
Crizotinib	0.14	0.52	0.31	0.03
Chemotherapy	0.76	0.18	0.05	0.01

DCR, disease control rate; ORR, overall response rate; PFS, progression‐free survival.

In terms of the ORR, the response rates were significantly higher for ALK inhibitors than for chemotherapy [OR and corresponding 95% CI: alectinib, 11.69 (4.29‐36.56); ceritinib, 7.85 (3.44‐19.27); crizotinib, 6.04 (3.33‐11.71)] (Table 2B). Ceritinib had a higher ORR than crizotinib, although the difference was not statistically significant [OR and corresponding 95% CI: 1.31 (0.45‐3.78)]. Rank probabilities also confirmed that the ORR of alectinib was the best among the four treatments; the ORR of ceritinib was likely the second best, and that of chemotherapy was the worst among them (Table 3B).

For DCR, the rank order was similar to that of ORR (Table 3C), although no significant differences were found among the treatments (Table 2C).

In terms of safety, the discontinuation rates were lower for ALK inhibitors than for chemotherapy [OR and corresponding 95% CI: alectinib, 0.42 (0.12‐1.36); ceritinib, 0.52 (0.20‐1.35); crizotinib, 0.70 (0.30‐1.62)] (Table 2D). This finding is also corroborated by the rank probabilities: chemotherapy ranked first in terms of the discontinuation rate, crizotinib ranked second, and alectinib was the safest among the four treatments (Table 3D).

The PSRF value was 1.00 for every model in this analysis, indicating that all models converged completely. Coherence between pairwise meta‐analyses and Bayesian meta‐analyses based on networks was confirmed.

### 3.3 Outcome evaluation and meta‐analysis

**Figure 3:**
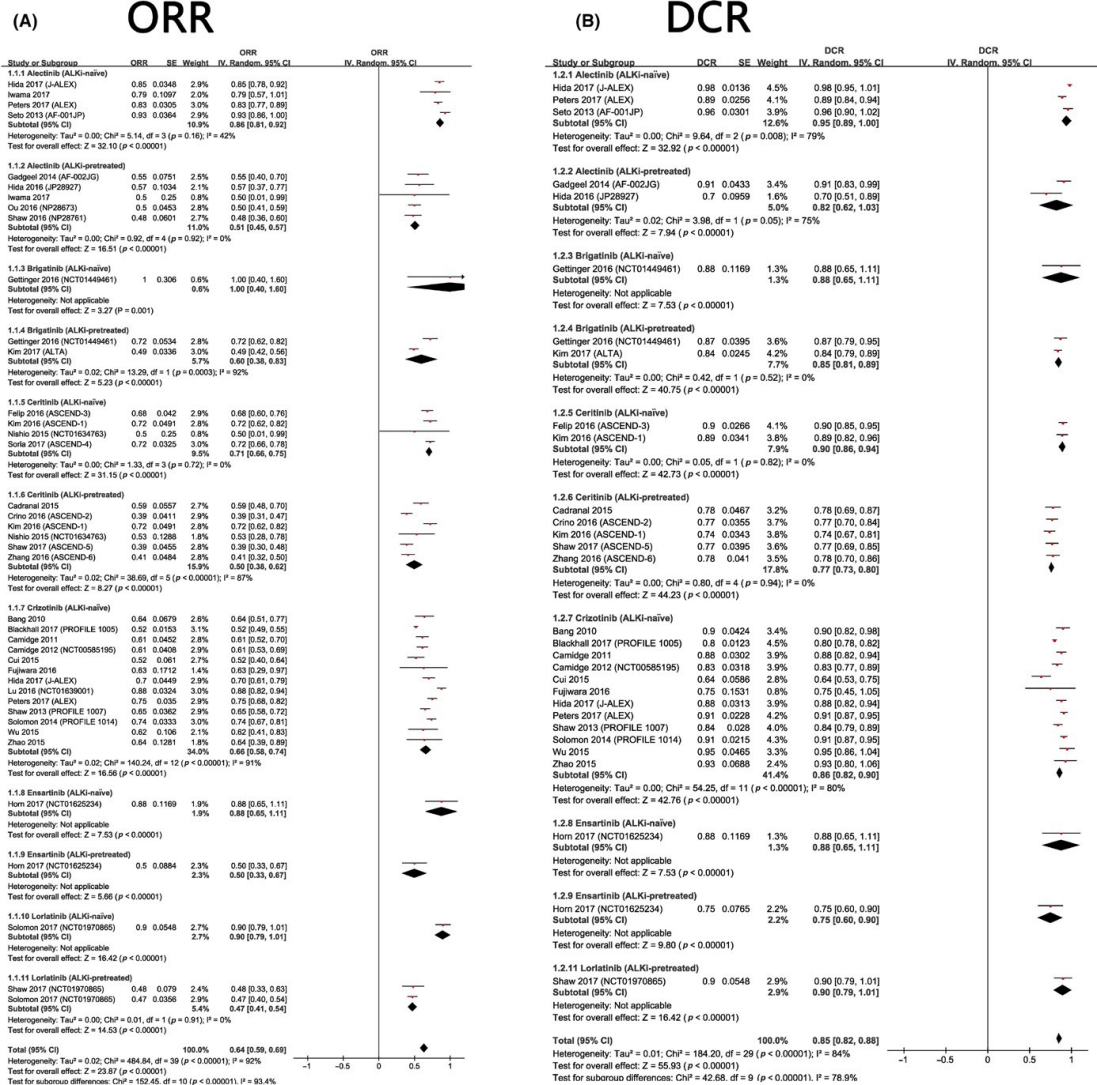
A, Meta‐analysis of the ORR of ALK+ NSCLC treated with ALK inhibitors. B, Meta‐analysis of the DCR of ALK+ NSCLC treated with ALK inhibitors

The aggregated ORR of ALKi‐treated NSCLC patients harboring ALK rearrangement was 64% (95% CI: 59%‐69%), and the pooled DCR was 85% (95% CI: 82%‐88%). For each ALKi, patients who have never received ALK inhibitors are more likely to response better than those who have received ALKi treatment before. In terms of the pooled ORR, ALKi‐naïve patients tended to respond better to third‐generation ALK inhibitors (brigatinib: 100%, ensartinib: 88%, lorlatinib: 90%) than second‐generation ALK inhibitors (alectinib: 86%, ceritinib: 71%) and first‐generation ALKi (crizotinib: 66%). The pooled DCR and pooled ORR of ALKi‐pretreated patients also had similar patterns (Figure 3).

The pooled PFS was 9.20 months (95% CI: 8.18‐10.22 months). For ALKi‐pretreated patients, those who were treated with brigatinib (12.51 months, 95% CI: 9.39‐15.63 months) and lorlatinib (10.00 months, 95% CI: 3.39‐16.61 months) tended to survive longer than those patients treated with alectinib (8.90 months, 95% CI: 6.77‐11.02 months) and ceritinib (6.42 months, 95% CI: 5.80‐7.03 months). For ALKi‐naïve patients, those who were treated with ceritinib (17.81 months, 95% CI: 13.40‐22.22 months) were more likely to survive longer than their crizotinib‐treated counterparts (9.47 months, 95% CI: 8.46‐10.49 months) (Figure 5).

**Figure 4:**
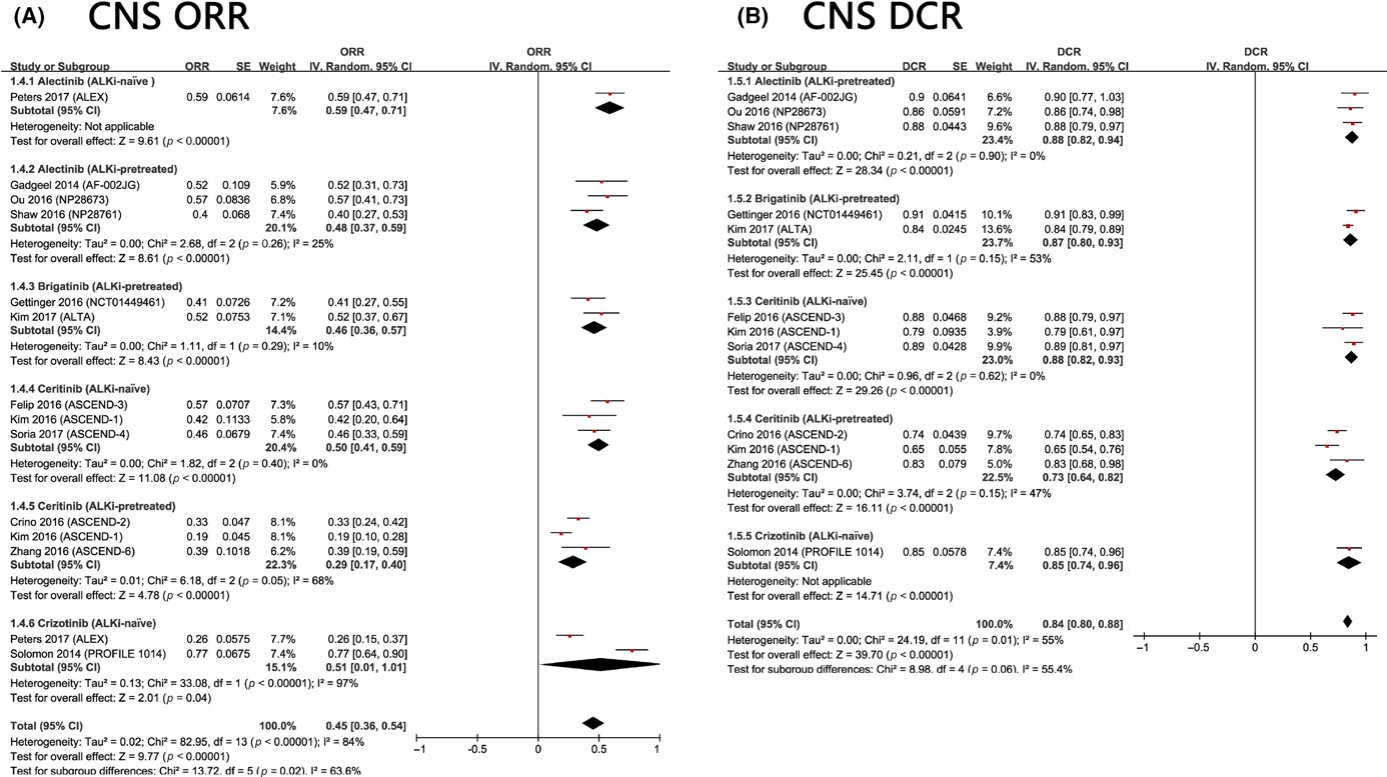
A, Meta‐analysis of the ORR of ALK+ NSCLC with baseline brain metastases. B, Meta‐analysis of DCR of ALK+ NSCLC with baseline brain metastases

Regarding the efficacy of ALK inhibitors in patients with brain metastases at baseline, the pooled intracranial ORR was 45% (95% CI: 36%‐54%), and the pooled intracranial DCR was 84% (95% CI: 80%‐88%). Likewise, patients who have never received ALK inhibitors are more likely to response better than those who have received ALKi treatment before. For ALKi‐pretreated patients, those treated with alectinib (48%, 95% CI: 37%‐59%) and brigatinib (46%, 95% CI: 36%‐57%) tended to have a higher ORR than those treated with ceritinib (29%, 95% CI: 17%‐40%). The pooled intracranial DCR also had a similar pattern. Notably, there was a remarkable discrepancy between the studies of Peters et al^9^ and Solomon et al^44^ regarding the intracranial ORR of crizotinib. Perhaps more patients with brain metastases need to be enrolled in future clinical trials to obtain better results to evaluate the intracranial efficacy of crizotinib (Figure 4).

**Figure 5:**
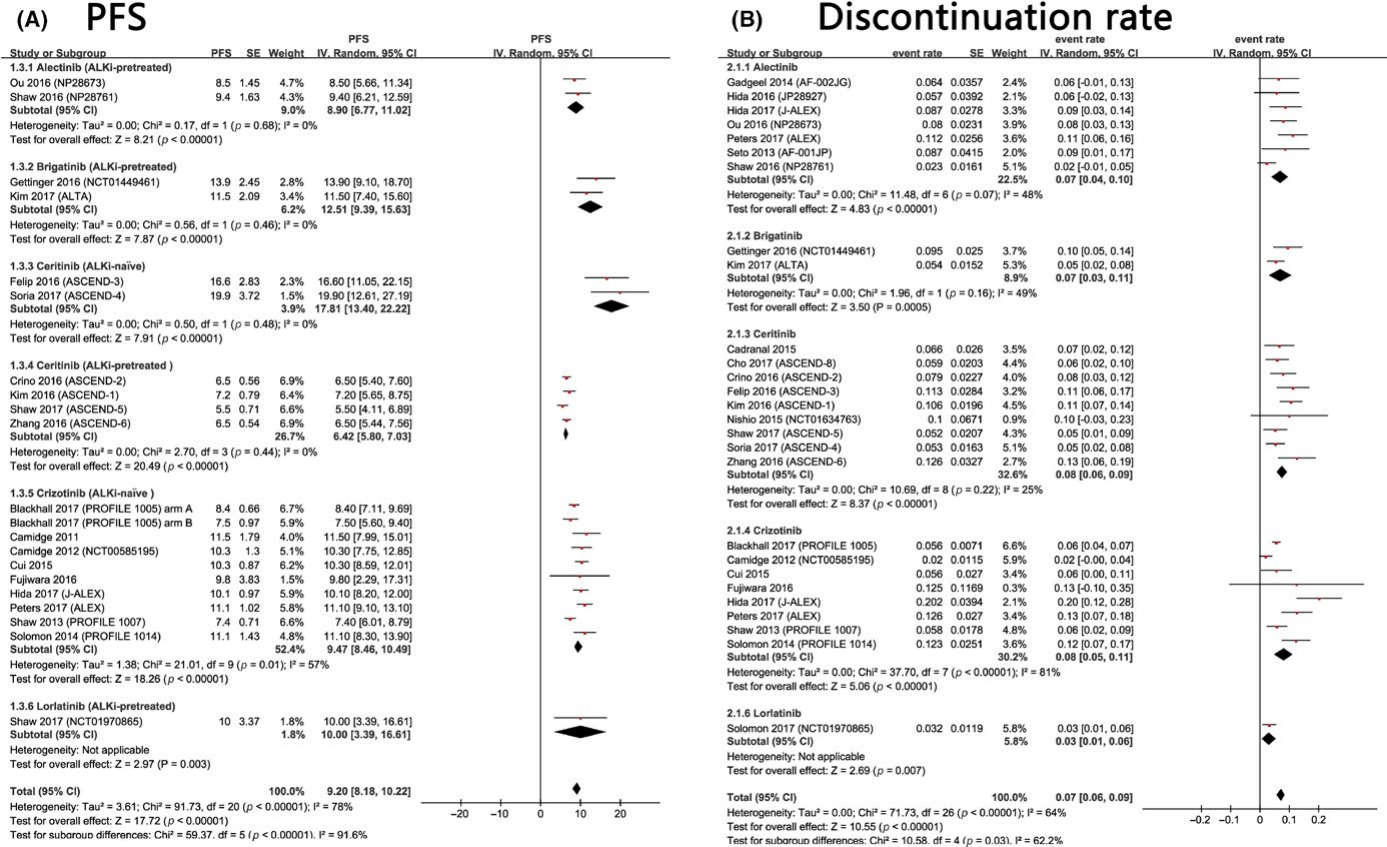
A, Meta‐analysis of the PFS of ALK+ NSCLC treated with ALK inhibitors. B, Meta‐analysis of the discontinuation rate due to adverse events of ALK inhibitors‐treated ALK‐rearranged non‐small cell lung cancer

The pooled discontinuation rate was 7% (95% CI: 6%‐9%). Subgroup analyses indicated that approximately 8% of ceritinib‐treated patients (95% CI: 6%‐9%) and crizotinib‐treated patients (95% CI: 5%‐11%) discontinued treatment due to various AEs, whereas 7% of alectinib‐treated patients (95% CI: 4%‐10%) and brigatinib‐treated patients (95% CI: 3%‐11%) required permanent discontinuation. Only 3% (95% CI: 1%‐6%) of lorlatinib‐treated patients needed to discontinue this treatment permanently (Figure 5).

### 3.4 Publication bias

The funnel plots for the ORR, DCR and intracranial ORR and DCR were roughly symmetrical (Figure S1). Egger and Begg tests also confirmed that publication bias was not significant (Table S1). However, the funnel plots for the pooled PFS and discontinuation rate were less symmetrical (Figure S1). Begg's and Egger's tests also yielded significant results (PFS: Egger's test, *P* < 0.001, Begg's test, *P* = 0.014; Discontinuation rate: Egger's test, *P* = 0.001, Begg's test, *P* = 0.003). These results indicated the existence of potential publication bias.

### 3.5 Survival analysis

**Figure 6:**
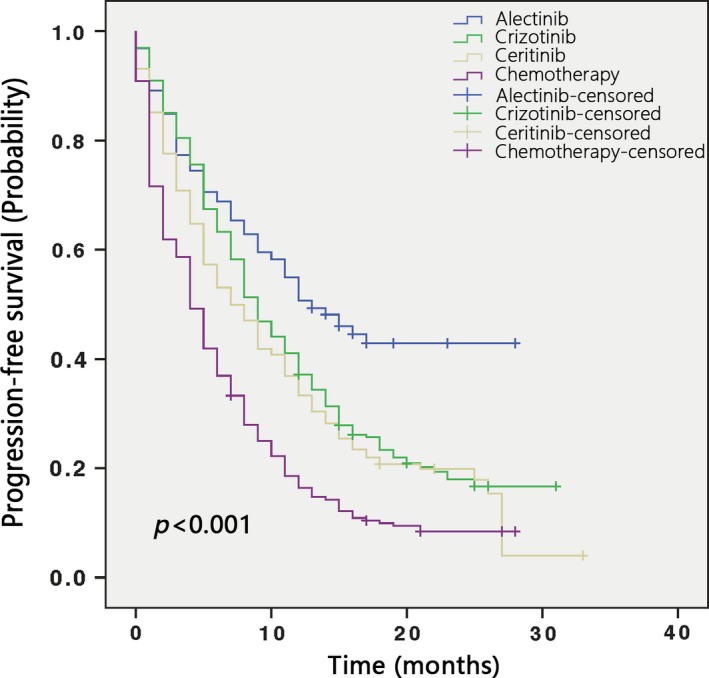
Kaplan‐Meier curves of PFS of ALK+ NSCLC treated with alectinib, ceritinib, crizotinib and chemotherapy

Pooled Kaplan‐Meier curves for PFS are displayed in Figure 6. The estimated median PFS and the corresponding 95% CI were as follows: alectinib, 13.0 months (11.107‐14.893 months); crizotinib, 9.0 months (8.250‐9.750 months); ceritinib, 7.0 months (6.118‐7.882 months); and chemotherapy, 4.0 months (3.543‐4.457 months; Figure 6).

## 4 DISCUSSION

Crizotinib was the first FDA‐approved ALKi for NSCLC patients.^45^ However, most patients develop drug resistance after approximately 10.5 months of crizotinib treatment.^7^ This acquired drug resistance prompted the development of second‐ and third‐generation ALK inhibitors. Nevertheless, few RCTs have been carried out to compare the efficacy and safety of different ALK inhibitors directly.^9^, ^10^ In the present study, we attempted to compare each ALKi by using both single‐arm and network meta‐analyses.

We found that compared with conventional chemotherapy, ALK inhibitors may significantly prolong patient PFS (Table 2A). Alectinib appeared to be more efficacious than ceritinib and crizotinib at prolonging PFS. Network meta‐analyses showed that the second‐generation ALKi ceritinib was even less efficacious than the first‐generation ALKi crizotinib, although the difference was not statistically significant [HR and 95% CI: 1.07 (0.95‐1.20)] (Table 2A). This finding was further corroborated by the results of a Kaplan‐Meier analysis (Figure 6). However, this result was contrary to the results of single‐arm meta‐analyses, in which the pooled PFS of ALKi‐naïve patients treated with ceritinib was 17.81 months, and the pooled PFS of their crizotinib‐treated counterparts was 9.47 months (Figure 5). Such discrepancies may result from differences in the baseline characteristics of the clinical trial participants. Therefore, head‐to‐head clinical trials comparing ceritinib and crizotinib are warranted to obtain a definitive conclusion.

Regarding the ORR and DCR, alectinib, and ceritinib were clearly better than crizotinib and chemotherapy, although the differences between ALK inhibitors were not statistically significant (Tables 2 and 3). We also found that for each treatment, ALKi‐naïve patients were more likely to have a better response than their ALKi‐pretreated counterparts (Figures 3 and 4). For patients with similar baselines (ALKi‐naïve or ALKi‐pretreated), third‐generation ALK inhibitors (lorlatinib, ensartinib, brigatinib) appeared to be more efficacious than second‐generation ALK inhibitors (alectinib, ceritinib) and the first‐generation ALKi crizotinib in terms of ORR (Figures 3 and 4).

For ALKi‐pretreated patients with baseline brain metastases, the second‐generation ALKi alectinib outperformed ceritinib and the third‐generation ALKi brigatinib for intracranial ORR and DCR. Similar patterns were also found in ALKi‐naïve patients. Intriguingly, the intracranial ORR of crizotinib in Peters et al's^9^ study was 26%, whereas in Solomon et al's^44^ study, it was 77%. The fluctuation in ORR results may be explained by the limited sample sizes. Further trials with larger sample sizes are warranted to investigate the intracranial effectiveness of crizotinib. In addition to the innate efficacy of each drug, the intracranial ORR may also be influenced by their ability to penetrate the blood‐brain barrier (BBB). An in vivo study has shown that alectinib, with a brain‐to‐plasma ratio ranging from 0.63 to 0.94, can penetrate the BBB well.^46^ However, another case report suggested that crizotinib, with a cerebral spinal fluid (CSF)‐to‐plasma ratio of 0.0026, has poor CSF penetration.^47^ The underlying mechanism may be closely associated with a protein called P‐glycoprotein (P‐GP) that is expressed in the BBB. Crizotinib and ceritinib can be effectively pumped out by P‐GP, but alectinib and lorlatinib are not substrates of P‐GP.^48^ P‐GP overexpression may mediate crizotinib resistance, and ceritinib and P‐GP inhibitors can help to overcome such resistance.^48^ Few studies have investigated the CNS penetration of each ALKi, so further studies are warranted to reveal the specific mechanism. This information may be helpful for developing novel ALK inhibitors with high intracranial efficacy.

Some patients have various AEs after anticancer therapies, and some may need to discontinue these treatments permanently. A lower discontinuation rate often indicates that the treatment is much safer. The network meta‐analyses proved that the discontinuation rates were lower for alectinib and ceritinib than for crizotinib and chemotherapy; however, the differences between each treatment were not statistically significant (Tables 2 and 3). Single‐arm meta‐analyses showed that the discontinuation rates were lower for alectinib and brigatinib (7%) than for ceritinib and crizotinib (8%), and lorlatinib had the lowest discontinuation rate (3%) (Figure 5). Some previous studies have analyzed the safety of crizotinib and alectinib.^12^, ^13^ Next‐generation ALK inhibitors appear to have better safety profiles. However, some parameters, including the proportion of people who need dose reduction or interruption, are not reported in all studies, and there are limited numbers of published studies of some next‐generation ALK inhibitors (lorlatinib, ensartinib, etc.).^2^, ^3^, ^49^, ^50^ Additional studies with more detailed data are warranted to analyze the safety of different ALK inhibitors comprehensively.

Nevertheless, our current research may have some limitations. Most of the included studies are phase 1 or 2 trials. Only eight random controlled trials were included. Therefore, we have only four nodes in our network meta‐analysis, and some analyses could not be performed due to the relatively simple structure of our network. Moreover, as displayed in Table 1, some included studies used different doses of treatment, this would also contribute to heterogeneity. Additionally, some of the baseline parameters of enrolled patients, as well as follow‐up times, were different in each study, which may also influence the results. More head‐to‐head random controlled trials are warranted to enrich the network and update our meta‐analysis.

In summary, our current analyses found that ALKi‐treated ALK+ NSCLC patients tend to have a longer PFS than their chemotherapy‐treated counterparts. Patients treated with alectinib or ceritinib are more likely to have a higher ORR and DCR than crizotinib or chemotherapy‐treated patients with similar baselines. For each treatment, patients who have never received ALK inhibitors are more likely to response better than those who have received ALKi treatment before. For NSCLC brain metastases, alectinib is preferable for its relatively high intracranial efficacy. In terms of safety, the discontinuation rates were lower for alectinib and ceritinib than for crizotinib and chemotherapy, although no statistically significant differences were found among the treatments.

## CONFLICT OF INTEREST

The authors declare that they have no conflict of interest.

## INFORMED CONSENT

This article does not contain any studies with human participants or animals performed by any of the authors. And therefore, no informed consent was involved.
