# Supplementary figures and images for: The efficacy and safety of ALK inhibitors in the treatment of ALK‐positive non‐small cell lung cancer: A network meta‐analysis
Source: Cancer Med. 2018 Sep 19;7(10):4993–5005. doi: 10.1002/cam4.1768 (PMC6198244; doi:10.1002/cam4.1768)

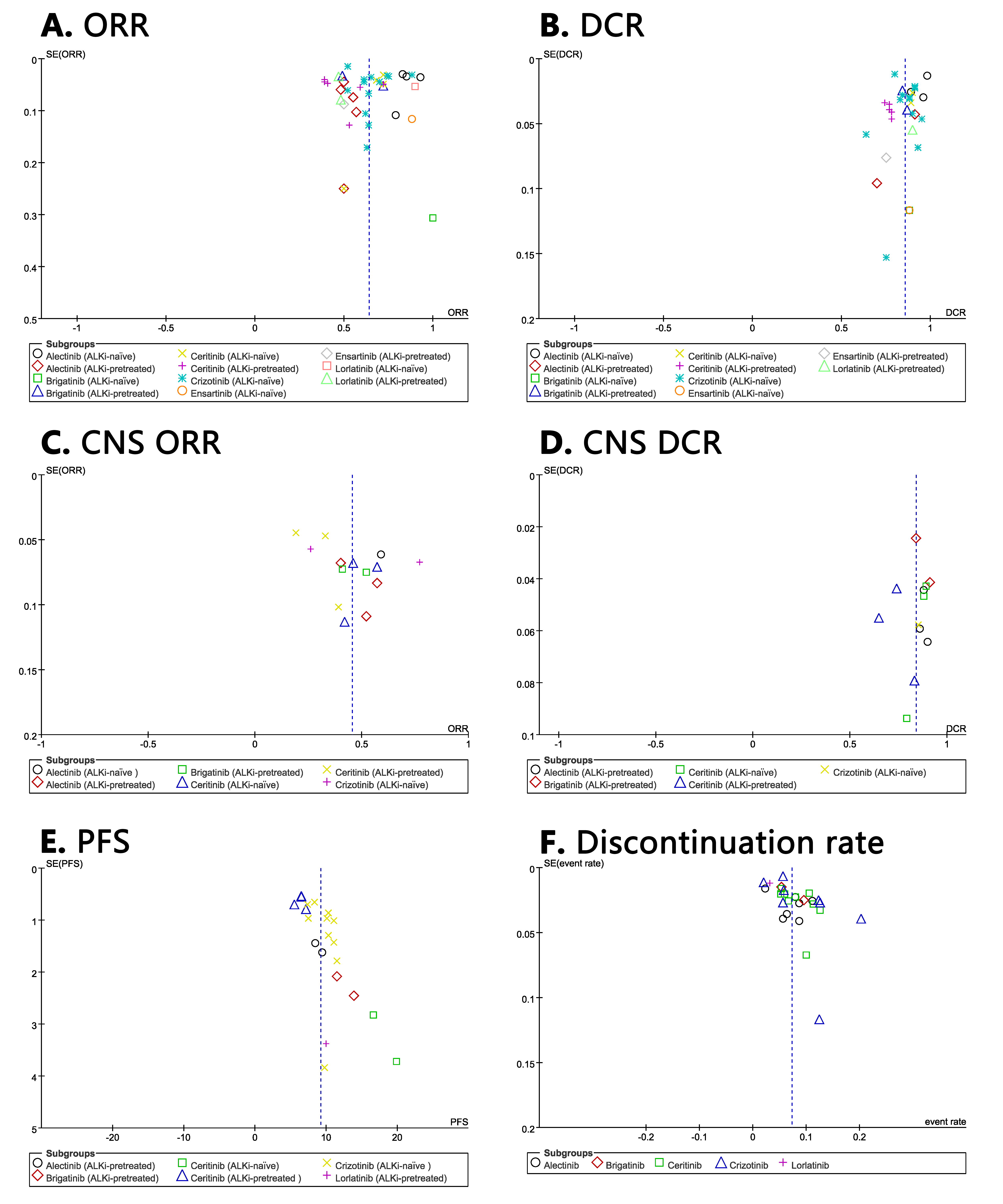

Supplement: Supplementary file 1 [file CAM4-7-4993-s001.tif]
