# Supplementary material for: The efficacy and safety of ALK inhibitors in the treatment of ALK‐positive non‐small cell lung cancer: A network meta‐analysis
Source: Cancer Med. 2018 Sep 19;7(10):4993–5005. doi: 10.1002/cam4.1768 (PMC6198244; doi:10.1002/cam4.1768)
Supplement: Supplementary file 2 [file CAM4-7-4993-s002.docx]

**Table S1. Begg’s and Egger’s test for all the results.**

|  | **Begg’s test** | **Egger’s test** |
| --- | --- | --- |
| Efficacy |  |  |
| ORR | 0.477 | 0.707 |
| DCR | 0.153 | 0.234 |
| PFS | 0.014 | <0.001 |
| CNS ORR | 0.443 | 0.067 |
| CNS DCR | 0.244 | 0.718 |
| Safety |  |  |
| Discontinuation rate | 0.003 | 0.001 |

Abbreviations: ORR, overall response rate; DCR, disease control rate; PFS, progression-free survival; CNS, central nervous system.
